# Autophagy in the retinal pigment epithelium: a new vision and future challenges

**DOI:** 10.1111/febs.16018

**Published:** 2021-05-31

**Authors:** Daniela Intartaglia, Giuliana Giamundo, Ivan Conte

**Affiliations:** ^1^ Telethon Institute of Genetics and Medicine Pozzuoli (Naples) Italy; ^2^ Department of Biology University of Naples Federico II Naples Italy

**Keywords:** AMD, autophagy, lysosomal storage disease, mTOR, retinal pigment epithelium

## Abstract

The retinal pigment epithelium (RPE) is a highly specialized monolayer of polarized, pigmented epithelial cells that resides between the vessels of the choriocapillaris and the neural retina. The RPE is essential for the maintenance and survival of overlying light‐sensitive photoreceptors, as it participates in the formation of the outer blood–retinal barrier, phagocytosis, degradation of photoreceptor outer segment (POS) tips, maintenance of the retinoid cycle, and protection against light and oxidative stress. Autophagy is an evolutionarily conserved ‘self‐eating’ process, designed to maintain cellular homeostasis. The daily autophagy demands in the RPE require precise gene regulation for the digestion and recycling of intracellular and POS components in lysosomes in response to light and stress conditions. In this review, we discuss selective autophagy and focus on the recent advances in our understanding of the mechanism of cell clearance in the RPE for visual function. Understanding how this catabolic process is regulated by both transcriptional and post‐transcriptional mechanisms in the RPE will promote the recognition of pathological pathways in genetic disease and shed light on potential therapeutic strategies to treat visual impairments in patients with retinal disorders associated with lysosomal dysfunction.

## Graphical abstract

RPE cells have different systems (mitophagy, ER‐phagy, xenophagy, lipophagy, ribophagy, pexophagy, aggrephagy, and LAP) for breaking down and recycling damaged materials, allowing them to reduce ‘waste’ and reuse the ‘raw material’. Autophagosomes and phagosomes work as recycler and they take care of the cellular waste transport to the ‘incinerator’: the lysosome. This factory produces scrap molecular pieces through a powerful enzyme machinery to ensure the correct waste RPE disposal.

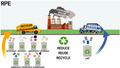

**Abbreviations**
11-cis RAL: 11-cis retinal
4EBP1: eukaryotic translation initiation factor 4E bind protein 1
AICAR: 5-Aminoimidazole-4-carboxamide-1-β-D-ribofuranoside
AMPK: AMP-activated protein kinase
ASB: arylsulfatase B
ATG12: autophagy-related 12
ATG13: autophagy-related 13
ATG14: autophagy-related 14
ATG16L1: autophagy-related 16-like 1
ATG2: autophagy-related 2
ATG3: autophagyrelated 3
ATG5: autophagy-related 5
ATG7: autophagy-related 7
Bcl‐2: B‐cell lymphoma 2
Bcl‐XL: B‐cell lymphoma‐extra large
BECN1: beclin 1
Cryba1: crystallin beta A1
CTSA: cathepsin A
CTSD: cathepsin D
DS: dermatan sulfate
FIP200: family‐interacting protein of 200 KDa
GBA: glucocerebrosidase
GLA: α‐Galactosidase A
GNPTAB: N‐acetylglucosamine‐1‐phosphate transferase‐α subunit
GNPTG: N‐acetylglucosamine‐1‐phosphate transferase‐γ subunit
GUSB: β‐Glucuronidase
HEX: hexosaminidase
HS: heparan sulfate
LC3B: microtubule‐associated protein 1 light‐chain 3 beta
MAN2B1: alpha‐mannosidase
MCOLN1: mucolipin‐1
mTOR: mechanistic target of rapamycin
mTORC1: mechanistic target of rapamycin complex 1
NCL: neuronal ceroid‐lipofuscinosis
NEU1: neuraminidase
ONL: outer nuclear layer
P150: chromatin assembly factor 1, subunit A
PARK2: parkin 2
PI3KCIII: phosphatidylinositol‐3‐kinase class III
PIP(3)K: phosphatidylinositol‐3‐kinase
PtdIins3P: phosphatidylinositol‐3‐phosphate
PUFA: polyunsaturated fatty acids
Rab9: ras‐related protein Rab‐9
RB1CC1: RB1‐inducible coiled‐coil 1
Rheb: Ras homolog enriched in brain
S6K: S6 KINASE
SGSH: N‐sulphoglucosamine sulphohydrolase
TFEB: transcription factor EB
TRPM1: transient receptor potential cation channel subfamily M member 1
TSC1: tuberous sclerosis protein 1
TSC2: tuberous sclerosis protein 2
ULK1: UNC51‐like kinase
Vps34: vacuolar protein sorting 34
WIPI1: WD repeat domain, phosphoinositide‐interacting 1

## Introduction

Autophagy is an evolutionary conserved catabolic process used by eukaryotic cells to degrade and recycle cytoplasmic proteins, damaged organelles, and other cellular constituents, playing an essential role in the maintenance of cellular homeostasis. Three major forms of autophagy have been described in cells: macroautophagy, chaperone‐mediated autophagy, and microautophagy. Extensive studies have shed light on the molecular mechanisms behind these processes, and our understanding of them is constantly updated with new discoveries. Macroautophagy (hereafter called autophagy) begins with the formation of double‐membrane vesicles, called autophagosomes, which surrounds cellular components destined for degradation; autophagosomes then fuse with lysosomes and sequestered components are degraded by acidic hydrolases, each capable of degrading a specific substrates [1]. Although in the past autophagy has been mostly considered a nonselective degradative process, an increasing amount of evidence have demonstrated that autophagy selectively degrades potentially harmful intracellular material such as mitochondria (mitophagy), the endoplasmic reticulum (ERphagy), peroxisomes (pexophagy), ribosomes (ribophagy), pathogens (xenophagy), liposomes (lipophagy), and aggregated proteins (aggrephagy). Moreover, autophagy has been shown to be a supervisor of DNA damage and genomic instability. Studies have focused on the molecular mechanisms behind these processes highlighting the importance of autophagy receptors and key adaptor proteins, which link substrates to autophagy machinery. So far, a number of landmark papers have reviewed autophagy in different fields including selective cargo recognition and degradation [2, 3]; transcriptional and post‐transcriptional regulation of autophagy [4, 5]; dynamic molecular mechanisms participating in the formation of core autophagic machinery and its fusion with lysosomes [6, 7, 8, 9, 10, 11, 12, 13, 14, 15, 16, 17, 18, 19, 20, 21, 22, 23, 24, 25, 26, 27, 28]; and related processes governing final cargo degradation [17, 29]. The initiation of autophagy requires a UNC51‐like kinase (ULK1) that forms a complex with autophagy‐related 13 (ATG13) and family‐interacting protein of 200 KDa (FIP200), driving autophagosomes formation. Autophagy is often regulated by AMP‐activated protein kinase (AMPK)‐driven mechanisms; indeed, under various physiological and pathological conditions, activated AMPK stimulates ULK1 activity, as well as class III phosphatidylinositol‐3‐kinase (PI(3)K/Vps34) and Beclin 1 (BECN1), leading to induction of autophagy. Interestingly, AMPK‐mediated pathways do not only bring about positive regulation of autophagy, but are also related to mechanistic target of rapamycin (mTOR) pathway inhibition. On one side, AMPK phosphorylates tuberous sclerosis protein 2 (TSC2) inducing Rheb‐mediated GAP activity, while on the other side AMPK drives Raptor phosphorylation, leading to mechanistic target of rapamycin complex 1 (mTORC1) inactivation and autophagy induction [30]. Furthermore, Qin *et al*. showed that AMPK activation, due to oxidative stress conditions, results in retinal pigment epithelium (RPE) phagocytosis inhibition. In particular, the authors demonstrated that blocking AMPK, through its activator 5‐aminoimidazole‐4‐carboxamide‐1‐β‐D‐ribofuranoside (AICAR), in RPE cells fully re‐established RPE phagocytosis. Moreover, knockout of the *AMPK* α2 isoform revoked oxidative stress‐induced inhibition of RPE cell phagocytosis [31].

**Fig. 1:**
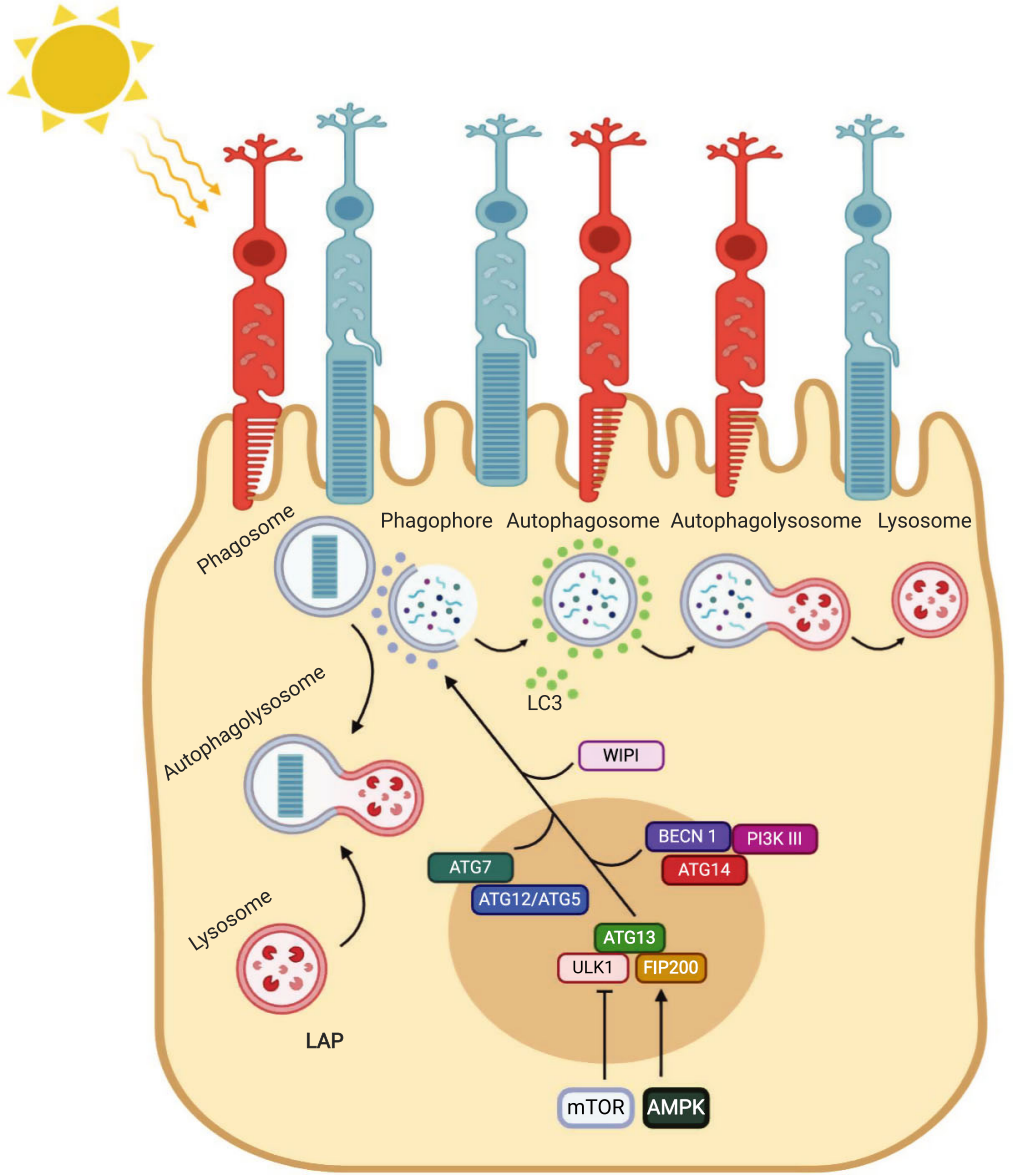
The two main autophagy pathways in RPE cells. Simplified schematic of the network regulation to ensure the correct formation of all the components that take part in autophagy and LAP pathways.

Initiation of autophagy requires the BECN1–Vsp34–ATG14‐p150 complex to mediate the synthesis of PtdIns3P, which in turn recruits WD repeat domain, phosphoinositide‐interacting 1 (WIPI1) and autophagy‐related 2 (ATG2) in the formation of a nascent autophagosome. Elongation and closure of the autophagosome are mediated by an autophagy‐related 7 (ATG7)‐dependent autophagy‐related 12 (ATG12)/autophagy‐related 5 (ATG5) conjugation system, which in turn is responsible for conjugation of phosphatidyl ethanolamine to the microtubule‐associated protein 1 light‐chain 3 beta, also known as LC3B [32]. Finalization of autophagosome vesicles ensures inclusion of the cargo, which is relayed after the fusion event between the autophagosome and lysosome, into a structure named the ‘autophagolysosome’ as recently reviewed elsewhere [33, 34, 35]. Whereas autophagy occurs in all cells, a specialized noncanonical form of autophagy, namely LC3‐associated phagocytosis (LAP), has recently emerged as a major mechanism in the RPE for the degradation of shed photoreceptor outer segment (POS) and the regeneration of part of the retinoid, responsible for the synthesis of the chromophore 11‐cis retinal (11‐cis RAL), thus supporting both disk renewal and the visual cycle (Fig. 1) [36]. Importantly, LAP is not dependent on the AMPK/mTORC1/ULK1 axis and does not seem to be stimulated by nutrient status or intracellular stress sensing [37, 38, 39, 40]. In the RPE, nascent phagosome formation, which precedes LAP, is regulated by the same molecular mechanisms that control phagocytosis. However, immediately after phagosome initiation, LAP exploits Vps34/BECN1 and the autophagy components ATG5, ATG7, ATG3, ATG12, and autophagy‐related 16‐like 1 (ATG16L1) for the recruitment and lipidation of LC3 at the single membrane phagosomes. Therefore, since LAP and autophagy share several molecular mediators, such as the class PI3KCIII, LC3, BECN1, and both ATG5 and ATG7 proteins, it is not surprising that these processes compete for the same resources and must be strictly controlled to ensure the efficiency of degradative processes in RPE cells and to avoid disrupting the cellular homeostasis [36, 41]. In this context, continual replenishment of lysosomes is required to replace those which are consumed in autophagy processes; this renewal process is critical to maintain optimal levels of cell clearance [42]. Importantly, most of the molecular events implicated in lysosomal biogenesis are controlled by both light stimuli and circadian rhythms. On a daily basis (every morning just after lights‐on), lysosomes are involved in LAP to degrade the phagocytosed shed POS. Additionally, lysosomes are employed in canonical autophagy to efficiently remove cellular waste in response to oxidative stresses and light, which are constantly imposed on the RPE cells. A failure in any one of these processes would result in an accumulation of toxic debris that will irreparably harm the RPE cells by pushing them toward cell death, supporting the idea that autophagy is critical to the health of the visual system. Recent studies highlighted the role of Rubicon in guaranteeing the proper balance between these two processes, as it plays a positive role in LAP and has a negative function in autophagy, as it prevents autophagosome maturation [40, 43]. Nevertheless, the role of additional modulators controlling autophagy and lysosomal biogenesis in the RPE is increasingly emerging and thus additional mechanisms on POS and/or autophagosomes delivery to the lysosomes cooperating with LAP and autophagy need to be explored. Consistently, different findings have highlighted a complex network between the proteasomal, lysosomal, autophagy, and exocytosis pathways necessary to ensure correct POS clearance [44, 45, 46, 47]. This review is focused on the most recent findings in the field, illustrating how the autophagy and lysosomal functions are exploited in the RPE under intracellular and extracellular stimuli in both healthy and disease conditions. In addition, we also discuss the challenges still to be addressed, namely how to improve the efficacy of autophagy to develop new therapeutic approaches to treat retinal diseases in which lysosomal functions are affected.

## Autophagy, stress stimuli, and cell survival in the RPE

Maintaining the correct level of autophagic activity in the RPE is highly relevant for the normal function of the visual system. In the RPE, selective autophagy is a very dynamic process that undergoes rapid activation under various physiological and pathophysiological conditions. Autophagy is part of an essential prosurvival program induced by a wide variety of stress stimuli including hypoxia, oxidative stress, nutrient and energy deprivation, ER stress, mitochondrial damage, and danger‐associated molecular patterns including light‐induced damage [48]. Importantly, the autophagic pathway in RPE cells changes and dysregulates during physiological aging and in disease conditions. Due to its function as a self‐adaptive stress–response process, deficiencies in the autophagic pathway can result in unsuccessful adaptation to stress conditions, which increases predisposition to cell death. Probably, the most extensively studied example of the crosstalk between autophagy and apoptosis is the molecular interaction of BECN1 with B‐cell lymphoma 2 (Bcl‐2) family members [49]. Several lines of evidence have demonstrated that the anti‐apoptotic factors, Bcl‐2 and B‐cell lymphoma‐extra large (Bcl‐XL), act as negative regulators of BECN1‐dependent autophagy [50]. Conversely, apoptosis can inhibit autophagosome formation by inducing the caspase‐mediated cleavage of BECN1 [51]. Importantly, the role of autophagy in the regulation of RPE survival has been largely studied using autophagy modulator‐deficient mouse models; for example, BECN1‐ and Parkin 2 (PARK2)‐deficient mice show an increased susceptibility to light‐induced retinal degeneration. BECN1 deficiency results in embryonic lethality, whereas BECN1^+/−^ mice displayed a loss of RPE and photoreceptors integrity during photo‐oxidative stress. Besides, PARK2 is a crucial component of the mitophagy machinery and its loss in mice‐induced typical features of light‐induced photoreceptor damage, but do not present any basal differences compared to PARK2^+/+^ mice [52]. These findings support the prosurvival role of autophagy as a protective mechanism against RPE cell death. Interesting data further suggest a key role for autophagy in preventing cell death in aging RPE. Studies have shown that genetic deletion of the essential macroautophagy gene *RB1 Inducible Coiled‐Coil 1* (*RB1CC1*) leads to inadequate handling of the ingested material, leading to age‐dependent RPE death and loss of photoreceptors [53]. In addition, in both *Atg5* and *Atg7* RPE‐conditional KO mice with reduced autophagy activity, RPE ‘clearing skills’ are impaired, leading to an Age‐related macular degeneration (AMD)‐like phenotype in aged RPE cells [54]. However, recent studies have also revealed that RPE‐specific deletion of *Atg5* or *Atg7* is not sufficient to induce retinal degeneration [36, 55, 56]. In fact, Kim *et al*. found that *Atg5*‐deleted RPE cells showed an alteration of lysosomal activity, resulting in accumulation of phagolysosomes containing poorly processed POS. Moreover, in these deficient cells, POS are located more apically than in control cells and phagosomes appear unable to cross the RPE to be degraded by lysosomes, proving that *Atg5* is critical to the phagocytotic process in the RPE. Nevertheless, the loss of autophagy caused by a deletion of *Atg5* in the RPE led an impairment in retinal function, without a significant decrease in photoreceptors number [36]. Furthermore, in mice lacking *Atg7*, Perusek *et al*. [55] reported a reduction in the removal of oxidized cellular components mediated by autophagy and an accumulation of vacuoles containing partially processed POS, although the retinal structure was preserved, together with a normal amount of the lipofuscin component A2E. In addition, the specific deletion of *Atg7* in Tyr‐Cre:*Atg7* mice resulted in the accumulation of both LC3‐I and p62, providing support for its role in autophagic flux suppression. These reduced autophagy levels produced increased expression of components of the regulatory network of the visual cycle [56]. These findings support the existence of an alternative *Atg5*/*Atg7*‐independent pathway, regulated by the factors of conventional autophagy, such as ULK1 and BECN1, or other proteins indirectly related to autophagy, such as RAB9 [57]. Therefore, further understanding of the molecular mechanisms by which autophagy controls RPE cell survival and death will accelerate the identification of therapeutic targets for counteracting RPE‐related diseases.

## Circadian and noncircadian autophagy in the RPE

Autophagy is central to RPE function and is tightly regulated under different metabolic and physiological conditions. Significant insights have been obtained by studying circadian‐ and light‐mediated activation of autophagy pathway in the RPE. [52, 58, 59, 60]. Firstly, Goldman *et al*. [61] demonstrated that autophagy is ruled by circadian rhythms; light pulses are able to regulate disk shedding and autophagy, which suggests that additional regulative mechanisms control these processes. Yao *et al*. [60] showed that autophagy activity is cyclically employed in the RPE and pursues a bimodal course during light/dark transitions, as measured by conversion of LC3‐I to LC3‐II; indeed, LC3‐II switch levels are linked to the time of day (Fig. 1). In particular, they and others [36] revealed that the cyclical nature of autophagy activation is in response to the circadian shedding of POS. The close linkage between autophagy machinery and POS phagocytosis is a valid support for visual function. Indeed, RPE cells must be constantly replaced; this happens thanks to daily phagosomal handling of POS and autophagy involved in the renewal of cellular components in RPE cells [62]. It has been estimated that each RPE cell phagocytoses hundreds of thousands of POS disks over a human lifetime [36]. Major components of the renewal mechanism follow a circadian rhythm [63] and thus represent a highly regulated process. Inner segment autophagic degradation also shows rhythmicity, which persists in constant light, but is rapidly abolished in constant darkness. In addition, both disk shedding and autophagy can be evoked by light pulses, further suggesting regulative functions of these processes. Moreover, evidence of circadian‐regulated autophagy is represented by the cyclic expression patterns of autophagy‐related proteins, including ATG5, ATG12, and ATG7 [36, 60]. Recently, Naso *et al*. [64] demonstrated that miR‐211 controls autophagy function at the beginning of light–dark transitions in the RPE by targeting *Ezrin*. Indeed, the molecular imbalance during the switch from dark to light conditions that occurs in miR‐211^−/−^ mice causes diurnal impairment of lysosomal biogenesis and autophagy together with an accumulation of phagolysosomes filled by undigested materials. *MiR‐211*‐mediated control of EZRIN induces an intracellular Ca^2+^ influx causing the activation of calcineurin, which in turn stimulates the nuclear translocation of transcription factor EB (TFEB), a master regulator of lysosomal biogenesis. The lack of *miR‐211* impairs light‐mediated induction of lysosomal biogenesis in the RPE, leading to age‐dependent compromised vision [64].

Autophagy peaks at the middle of the light phase (in cycles of 12‐h dark/12‐h light) and is thought to play a role in the degradation of visual pigments, which enables adaptation to new light conditions [65]. As such, autophagy‐mediated degradation has been proposed as a means of reducing the capacity for light absorption, thus protecting photoreceptor cells from light‐induced damage. Autophagy activity may be disturbed by various mechanisms during the degeneration of RPE cells and development of AMD. In aged RPE cells, persistent exposure to photooxidation exposes the RPE to oxidative stress leading to decreased proteolysis and lipofuscin accumulation in lysosomes [66, 67]. Accumulated intracellular lipofuscin and extracellular drusen deposits increase the probability of AMD development [68, 69, 70].

Although autophagy and phagocytosis can coexist in an interconnected manner in the RPE, they do not necessarily coincide and can occur independently. In the last decade, several studies have aimed to determine the interplay between LAP, phagocytosis, and POS degradation to improve vision. Cytoplasmic proteins are involved in the complex machinery that coordinates the fusion of phagocytic vesicles and lysosomes, thereby ensuring POS degradation. For example, in mice lacking *myosin VIIa*, RPE cells showed normal phagocytosis, but engulfed phagosomes remain blocked in the outer segment apical region and do not fuse with lysosomes [71]. Thus, the regulation of phagocytosis, in all its aspects, is critical to avoid the accumulation of POS in the RPE and to guarantee recycling of the visual components. Importantly, in the RPE both phagocytosis and lysosomal functions change with age and in disease conditions [72]. Age‐induced decreases in the latter cellular processes induce a pathological cascade that exacerbates autophagy misfunction, which leads to accumulation of oxidized lipids, defective mitochondria, and lipofuscin, followed by cellular dysfunction and retinal degeneration [73].

## RPE phenotype in LSDs

Tissue‐specific knockout studies in animal models (discussed above) highlight the key role of autophagy in RPE homeostasis and function for proper vision. Therefore, it is not surprising that many emerging studies have demonstrated how autophagy and lysosomal dysfunction are main players causing severe retinal diseases (Table 1) [74]. Indeed, defects in lysosomal proteins or lysosomal‐related proteins result in a group of heterogeneous inherited metabolic diseases such as lysosomal storage disorders (LSDs). Dysfunctional lysosomes show abnormal accumulations of toxic proteins, nucleic acids, carbohydrates, and lipids within all living cells, due to mutations in genes coding for specific lysosomal hydrolases. Nevertheless, some LSDs result from alterations in nonenzymatic proteins, such as lysosomal membrane proteins, involved in the transport of degradation materials [75]. The majority of LSDs share features such as impairment of autophagosome–lysosome fusion, reduced clearance of autophagic substrates, and deficient organelle recycling [76]. These diseases affect different cell types, tissues, and organs. Ocular complications occur as the early signs and symptoms of most LSDs and they underlie a prospective vision loss [77]. All parts of the eye may be affected in these storage diseases, such as cornea, lens, sclera, and retina. Different studies observed a progressive corneal opacification in the cornea of patients suffering from some LSD forms [78, 79]. Moreover, lens and sclera of these patients also show severe alterations, like bilateral peripheral cataracts and uveal effusions [78, 80]. Finally, the most common impairment in LSD eyes is retinopathy including RPE atrophy associated with visual field restriction [81]. One of the largest most studied group of LSD is the mucopolysaccharidoses (MPSs) characterized by the accumulation of glycosaminoglycans (GAGs) in the retina, RPE, cornea, and optic nerve, causing vision loss. Mutations in lysosomal genes lead to lysosomal impairments accompanied by GAG deposition within RPE cells and in the interphotoreceptor matrix, which in turn promotes photoreceptor loss [82], light sensitivity, and night blindness [78], all clinical signs also observed in retinitis pigmentosa [83]. In several MPS patients, electroretinography (ERG) examination revealed the severity in the progression of retinopathy, which appeared to be associated with a lack of rods rather than cones. On the other hand, additional clinical studies demonstrated that RPE pathology is not associated with defects in retinal structure and function [84]. Ocular manifestations in MPS are heterogeneous and a rapid and accurate evaluation of the eyes in patients with MPS may be challenging.

**Table 1:** Ocular dysfunctions in lysosomal storage diseases.

LSD type	Gene deficiency	Protein accumulation	Ocular phenotype	Reference
Tay–Sachs disease and Sandhoff disease	Hexosaminidase A (HEXA) and B (HEXB)	G_M2_ ganglioside	Destruction of rods and cones and vacuolated inclusions in RPE cells	[121]
Lysosomal acid lipase deficiency, Wolman disease, and cholesteryl ester storage disease	Acid lipase	Lipids	Inefficient elimination of POS debris and outer retinal degeneration	[122]
Neuronal ceroid‐lipofuscinosis	*CTSD* *CLN1‐8*	Sphingolipids Lipopigments	Retinal atrophy and photoreceptor degeneration	[123, 124]
Mucolipidosis	α‐Neuraminidase (*NEU1*), UDP‐N‐acetylglucosamine‐1‐phosphotransferase (*GNPTAB*, *GNPTG*), Mucolipin‐1 (*MCOLN1*)	Glycoproteins Carbohydrates Lipids	Severe retinal degeneration and POS incomplete degradation	[125, 126]
α‐Mannosidosis	α‐mannosidase (*MAN2B1*)	Mannose pentasaccharides	Incomplete degradation of rhodopsin containing outer segment material and RPE atrophy	[84, 127]
Galactosialidosis	Cathepsin A (*CTSA*)	Sialyloligosaccharides	Senescence of RPE	[128]
Gaucher disease	Glucocerebrosidase (*GBA*)	Glucosylceramide	Atrophic retina, photoreceptor, and RPE loss	[129]
Fabry disease	α‐galactosidase‐A (*GLA*)	Globotriaosylceramide	RPE cells apoptosis	[130]
Niemann–Pick disease	Acid sphingomyelinase	Sphingomyelin	Impaired visual function, lipofuscin accumulation in the RPE layer, degeneration of POS	[131, 132]
Metachromatic leukodystrophy	Arylsulfatase A	Sulfated compounds	RPE degeneration	[133]

MPS I is caused by a defect in the enzyme α‐L‐iduronidase leading to lysosomal accumulation of dermatan sulfate (DS) and heparan sulfate (HS). Retinal complications are common in children with MPS I; in fact, some of the young patients suffer from progressive rod‐cone degeneration associated with RPE layer alterations, while others show fibrillogranular inclusions in the RPE and defects in the retinal ganglion cells [85]. The cause of retinal abnormality in MPS I patients is a consequence of GAG accumulation in the RPE; however, the simultaneous Muller and RPE cells death may contribute to the pathogenesis of retinal diseases [86]. MPS II, also known as Hunter syndrome, is an X‐linked deficiency of lysosomal enzyme iduronate 2‐sulfatase that induces accumulation of GAGs in intracellular lysosomes. Mutations in this gene may contribute to a range of ocular abnormalities, as largely supported by the literature. A case report headed by Yamanishi *et al*. has pointed out ‘salt and pepper’ pigmentation of the RPE and reduced light sensibility of the retina with preservation of the visual‐evoked potentials [87, 88]. Optical coherence tomography (OCT) analysis mainly showed atrophy of the photoreceptors and RPE layers and, secondarily, an alteration of thickness of the ganglion cell layers and a mild raising of optic disks from the retina [88]. Sanfilippo syndrome, or MPS III, is the most common MPS class which results from defects in four different enzymes allowing classification into subtypes A‐D. In MPS III patients, undegraded HS accumulation in lysosomes results in a wide spectrum of clinical phenotypes, including significant visual impairment [78]. In particular, ERG and histopathology analysis have highlighted photoreceptors loss followed by severe retinal degeneration [89]. MPS IIIA is due to the lack of lysosomal sulfamidase N‐sulphoglucosamine sulphohydrolase (SGSH) that catalyzes the breakdown of HS. Importantly, Intartaglia *et al*. demonstrated the correlation between ocular pathology and lysosomal impairment in RPE cells in MPS IIIA mice. Observed lysosomal defects caused a strong microgliosis and a gradual loss of cone and rod density in MPS‐IIIA mouse retina [90]. MPS VI, Maroteaux–Lamy syndrome, is a lysosomal storage disorder caused by arylsulfatase B (ASB) deficiency. Several findings have demonstrated that impaired autophagy in MPS VI patients leads to vacuolated inclusions in the RPE, which is often associated with RPE hypertrophy and loss of the typical uniformity of the RPE layer [91]. The ASB deficiency involves a topographic variability in different regions of the RPE monolayer, according to varying lysosomal enzyme activity therein [92, 93]. As a consequence, hypertrophic RPE cells markedly lead to defects in adjacent POS and vision loss [94]. Finally, in the MPS VII patients, mutations in the β‐glucoronidase (GUSB) gene lead to a partial degradation of GAGs in the RPE, triggering a progressive retinal degeneration. The latter phenotype is mainly due to GAG accumulation in the RPE rather than to primary defects in the photoreceptor cells [95, 96], which are secondarily affected following a cone‐rod dystrophy [97]. Studies on animal models highlighted an age‐dependent decrease of ERG a‐ and b‐wave amplitudes in MPS VII mice from 6 weeks of age. Moreover, murine MPS VII retinas show a reduced length of rod outer segment, a prominent inflammation in the subretinal layer, together with a poorly organized outer nuclear layer (ONL), due to an accumulation of extracellular material storage and an increase of apoptosis levels [98]. Treatment of MPS disorders changes according on their seriousness and whether a combinatorial approach is needed. The most commonly adopted strategies either add a recombinant functional copy of the defective enzyme (enzyme replacement therapies—ERTs) or make use of viral vectors bearing the gene encoding the functional enzyme (gene therapies). Although promising results have been demonstrated, further studies are required to improve the efficacy of these strategies on retinal function and structure [99]. Moreover, due to impairment of the lysosomal‐autophagic pathway in MPSs, the current main research goal is the development of drug‐based alternatives to enhance the degradation of GAG aggregates to ameliorate MPS phenotypes [100].

## Autophagy, aging, and AMD

Autophagy and lysosomal function in the RPE cells changes with age and disease [101]. Several lines of study have demonstrated that impairments to the lysosomal degradative capacity in the RPE lead to progressive accumulations of lysosomal POS with their cargo and lipofuscin, which in turn results in RPE cell toxicity and pathological conditions [102]. The RPE is continuously exposed to oxidative stress due to diurnal digestion of polyunsaturated fatty acids (PUFAs) generated from phagocytosis of POS and light‐induced production of ROS. Even though the RPE protects the retina from oxidative stress, the RPE itself is sensitive to oxidative stress and protein accumulation because it is metabolically active, highly oxygenated, and exposed to photosensitizers such as the age‐pigment lipofuscin [58]. During the aging of RPE cells, the reduction of antioxidants occurs, this decreases the cellular ability to neutralize ROS, which in turn causes protein misfolding and accumulation of lipid and protein aggregates, accelerating RPE dysfunction [103] and thus leading to conditions such as AMD. It is therefore reasonable that autophagy is primary to guarantee RPE homeostasis, protecting it from oxidative damage and protein accumulation.

**Fig. 2:**
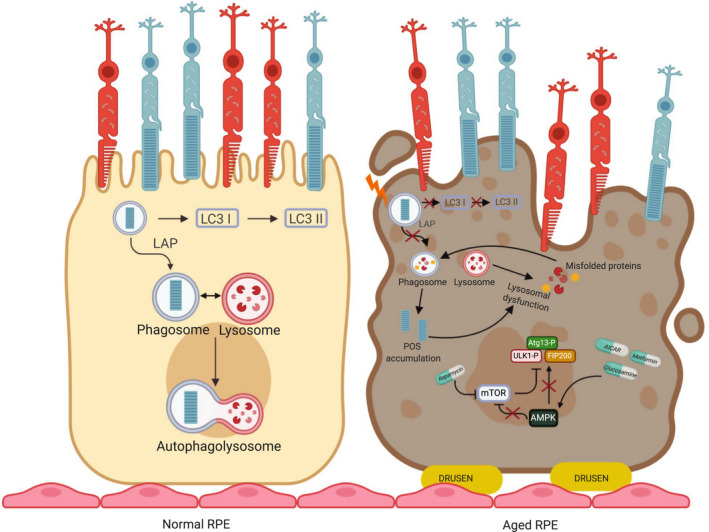
RPE autophagy dysfunction in AMD. Healthy RPE vs RPE in AMD. In normal RPE, an efficient autophagic flux and LAP processes occur. In aged RPE, oxidative stress and subsequent mTOR activation lead to lysosomal dysfunction and LAP impairment.

In the last decade, numerous studies have focused their attention on the relevance of autophagy in the RPE to prevent or slow‐down retinal diseases. AMD pathogenesis onset has been related to autophagic flux increase, which results in accumulation of damaged materials caused by aging‐induced ROS storage [52, 104]. Mitter *et al*., through RPE cell experiments, highlighted an oxidative stress dual effect on autophagy activity, based on exposure time: (a) long‐term exposure increases autophagy; (b) short‐term exposure decreases autophagy. On the other hand, they also observed a reverse relationship between autophagy and ROS generation. Indeed, aging‐related autophagy induction decreases oxidative stress, protecting RPE cells from damage and promoting cell viability, whereas autophagy impairment induces a substantial increase in ROS production, leading to improvements in cell death. Interestingly, RPE degeneration is correlated with improved mTORC1 activity [105]. Senescent RPE cells show an increased mTORC1 activation that leads to age‐related decay in autophagy function in the RPE. Importantly, several studies demonstrated that rapamycin‐mediated inhibition of mTORC1 and therefore activation of autophagy counteract senescence in RPE cells [106]. RPE cells contain both mTORC1 and mTORC2 complexes that can be functionally activated by different stimuli [106]. mTORC1 is useful for protein production and degradation in RPE cells. Specifically, under nutrient and growth factor stimulation, mTORC1 regulates protein synthesis through the phosphorylation of the downstream effectors S6K and eukaryotic translation initiation factor 4E bind protein 1 (4EBP1) [107]. In particular, activated mTORC1 phosphorylates ATG13, blocking its association with ULK1 and FIP200 employment [108, 109]. This condition decreases autophagy induction in RPE cells [110]. In the starvation state, mTOR is repressed, leading to dephosphorylation of ATG13 and resulting in autophagosome formation. Interestingly, mTORC1 exhibits rhythmic function following precisely controlled timing in response to the bursts of phagocytosis in RPE cells [106, 110]. Valapala *et al*. demonstrated that loss of *Crystallin Beta A1* (*Cryba1*), an essential component in the RPE required for autophagy and phagocytosis, leads to mTORC1 activation, thus impairing lysosomal function and autophagy in the RPE [111]. This suggests that *Cryba1* is crucial for mTORC1 signaling in the RPE. Furthermore, it has been showed that mice lacking *Cryba1*, specifically in the RPE, develop an AMD‐like pathology associated with defective lysosomal clearance [112]. Importantly, RPE‐specific deletion of the tuberous sclerosis protein 1 (*TSC1*) gene, encoding an upstream suppressor of mTORC1, abnormally increased the mTORC1 pathway leading to chronic accumulation of lipids and lipoproteins followed by RPE degeneration [113]. Moreover, Zhao *et al*., in their studies on primary cultured human RPE cells, found that metformin triggers AMPK signaling activation, which promotes autophagic flux allowing autophagosomes–lysosomes binding, and inhibits RPE cell damage [114]. Finally, several findings have demonstrated that glucosamine is able to stimulate AMPK activation *in vitro* and *in vivo* [115, 116]. Recent findings by Chen *et al*. showed that treatment of ARPE‐19 cells with glucosamine increased AMPK phosphorylation, decreased mTOR phosphorylation, and increased LC3‐II levels. Taken together, these results imply that glucosamine induces autophagy in RPE cells, via the AMPK‐mTOR signaling pathway [117] (Fig. 2). More recently, genomewide association study (GWAS), genetic, and eQLT data revealed *miR‐211* and its host gene transient receptor potential cation channel subfamily M member 1 (*TRPM1*) as possible causal genes for a new AMD locus [118, 119, 120]. These studies further supported the role for *miR‐211* in controlling TFEB‐mediated autophagy pathway in the RPE and highlighted the possible relevance of *miR‐211* in causing AMD‐like pathologies in mice. Notably, activators of autophagy seem to be effective as therapeutic approaches in *miR‐211^−/−^* mice, suggesting that proper modulation of lysosomal biogenesis and function might play a therapeutic role in enhancing protein degradation and ameliorating RPE cellular distress [64].

## Open issues

Overall, these data highlight the complexity of the autophagy pathway in retinal pigment epithelial cells, in which a perfect balance between canonical and noncanonical autophagy pathways must be retained. Multiple alternative molecular networks are emerging, which are necessary for the efficient removal of ingested POS from phagocytosis and for maintenance of RPE cellular homeostasis. Nevertheless, the vast heterogeneity of molecules involved in the control of the autophagy pathway suggests that the exact relationship between light, phagocytosis, and cell clearance warrants further exploration. Several questions remain: Which lysosomal proteins are required to mediate different autophagy pathways in the RPE? How are the different forms of autophagy connected? Do lysosomal proteins interact with each other by forming selective highly specialized complexes to remove phagocyted POS? Which specific aspects of autophagy are perturbed in RPE diseases? What is the molecular switch‐signal converting autophagy from cytoprotector to cytokiller? How are the different emerging molecular networks connected to lipofuscin accumulation and cumulative oxidative stress in aging RPE cells? Defects in autophagic genes represent a primary pathogenic cause for retinal degeneration. Targeting autophagy may prevent apoptosis, increase recycling of damaged organelles, promote mitophagy clearing of damaged mitochondria, and alleviate ROS‐induced oxidative stress. Therefore, the characterization of the underlying molecular networks is particularly relevant in view of developing new and more promising therapeutic approaches to counteract retinal diseases.

## Conflict of interest

The authors declare that the research was conducted in the absence of any commercial or financial relationships that could be construed as a potential conflict of interest.

## Author contributions

DI, GG, and IC wrote the manuscript and prepare the figures. All authors contributed to the article and approved the submitted version.
